# High-Sensitivity Cardiac Troponin Predicts Major Cardiovascular Events in Diabetic Patients With Critical Limb Ischemia and Foot Lesions

**DOI:** 10.3389/fcvm.2021.595701

**Published:** 2021-05-28

**Authors:** Paolo Cimaglia, Luca Dalla Paola, Anna Carone, Giuseppe Scavone, Marco Manfrini, Simona Brogneri, Elena Tenti, Rita Pavasini, Davide Bernucci, Giulia Passarini, Francesco Vitali, Eleonora Gaudenzi, Roberto Ferrari, Gianluca Campo

**Affiliations:** ^1^Cardiovascular Department, GVM Care and Research, Maria Cecilia Hospital, Cotignola, Italy; ^2^Cardiovascular Center, University of Ferrara, Ferrara, Italy

**Keywords:** biomarker, peripheral artery disease, diabetic foot, troponin, critical limb ischaemia

## Abstract

**Background:** Diabetic patients with critical limb ischemia (CLI) and foot lesions show a poor prognosis. Optimal risk stratification to guide tailored intervention is still uncertain. The aim of the present study was to assess the prognostic role of high-sensitivity cardiac troponin T (hs-TnT) in such a high-risk population.

**Methods and Results:** Clinical, laboratory, and interventional data, as well as the SPINACH score, were collected. Hs-TnT was measured at hospital admission. All patients were followed up for at least 1 year. The primary endpoint was the cumulative occurrence of major cardiovascular events (MACEs, all-cause death, myocardial infarction, or stroke). The secondary endpoint was all-cause mortality. Overall, 618 patients were included and followed for a median of 981 (557–1,325) days. Diagnosis of coronary artery disease (CAD) was established in 270 (43.7%) patients. Median hs-TnT at admission was 31 (20–59) ng/L, with 525 (85%) patients over the upper reference limit. Hs-TnT values were significantly higher in patients with established CAD (39 vs. 29 ng/L, *p* < 0.01). Hs-TnT was an independent predictor of MACE (HR 2.440, 95% CI 1.706–3.489, *p* < 0.001). The best cut-offs were 40 ng/L (AUC 0.711) for patients with established CAD and 25 ng/L (AUC 0.725) for those without. Hs-TnT emerged also as an independent predictor of all-cause mortality. The addition of hs-TnT improved prognostic value of the SPINACH score.

**Conclusions:** Hs-TnT is a powerful biomarker for prognostic stratification of diabetic CLI patients with foot lesions. This is confirmed independently to CAD diagnosis and permits the identification of higher risk patients requiring tailored intervention.

## Introduction

**Figure 1:**
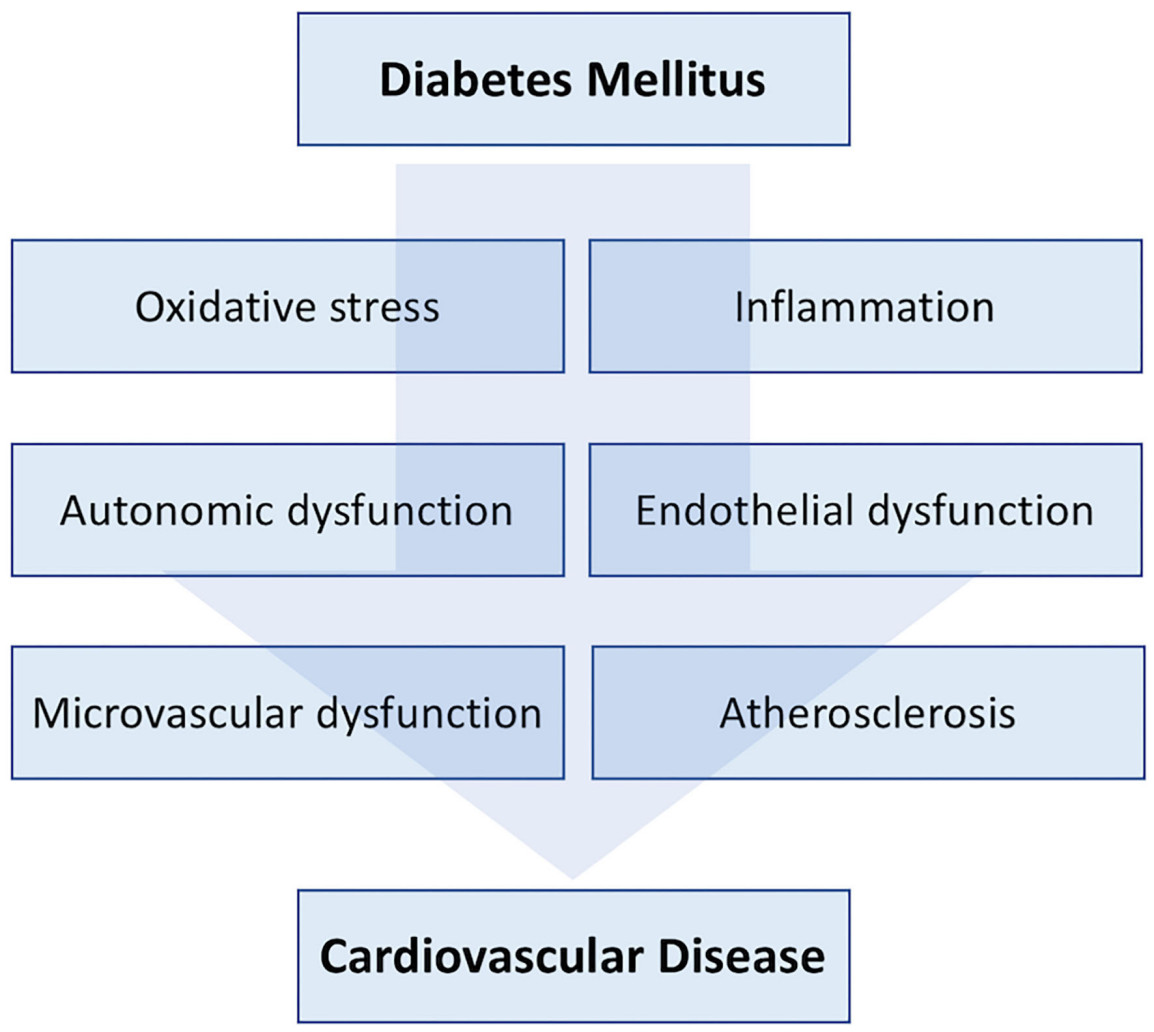
From diabetes mellitus to cardiovascular disease. Brief summary of pathophysiological mechanisms involved in the development of cardiovascular damage during diabetes mellitus.

Diabetes is a growing healthcare concern worldwide, and recent consensus documents estimated that up to 592 million people will be living with diabetes in 2035 (1). Diabetes is strongly associated with cardiovascular complications, including coronary artery disease (CAD) and peripheral artery disease (PAD), because it determinates micro- and macrovascular dysfunction through many pathophysiological mechanisms that comprehend inflammation, endothelium dysfunction, ion channels, and nervous system impairment (Figure 1) (2–5). Diabetic patients have a high chance to develop the most advanced form of PAD, namely, critical limb ischemia (CLI) with foot lesions (CLI with tissue loss or diabetic foot). Cardiac adverse events are common in these patients, independently of a concomitant diagnosis of CAD, with cardiac death being the most common cause of mortality (6, 7). The exacerbation of PAD or the need of procedures further increase the risk of cardiac adverse events (8).

The clinical management of these patients is challenging, regardless of whether patients are followed by experts of PAD or CAD or both. CAD patients with PAD are at higher risk compared with those without PAD, but the term “PAD” covers a wide range of conditions, and, in practice, PAD diagnosis does not lead to immediate management repercussions. Equally, for those patients with PAD and no CAD diagnosis, it is not known when and how it is appropriate to search for silent ischemia. To this end, the availability of reliable markers or score for stratification of risk of adverse events in diabetic patients with CLI and foot lesions is highly needed (9).

It has been suggested that cardiac troponin might be helpful for this purpose (10, 11). Cardiac troponin is a biomarker widely used in emergency settings for the diagnosis of acute coronary syndrome. In stable conditions, it has a role in the prediction of prognosis in several diseases, both cardiac (i.e., heart failure, pulmonary embolism, cardiac amyloidosis) and not (i.e., sepsis). The prognostic role of troponin in diabetic CLI patients with foot lesions has never been systematically determined. Previous studies in PAD patients at different stages did not stratify for concomitant established CAD diagnosis and did not measure high-sensitivity cardiac troponin (hs-Tn).

We aimed to fill this evidence gap. Accordingly, the prognostic role of hs-Tn in the prediction of adverse events has been investigated in a large, modern, prospective cohort of diabetic CLI patients with foot lesions, stratified for presence of CAD diagnosis, undergoing invasive procedures (lower limb angiography ± peripheral revascularization ± surgical treatment of foot lesions).

## Materials and Methods

Clinical and procedural data from all patients admitted to Diabetic Foot Unit of the Maria Cecilia Hospital (Cotignola, Italy) are recorded in a dedicated clinical database, verified for completeness and accuracy against the patients' clinical charts. Patients are prospectively followed up for at least 12 months. The analysis is based on current clinical practice; therefore, the regulatory authorities required an ordinary written informed consent to procedures and data collection, which is obtained from all patients. The protocol of the study is in accordance with the Declaration of Helsinki.

### Study Population

Inclusion criteria for the present analysis were (1) age >18 years, (2) diagnosis of diabetes mellitus, and (3) diagnosis of critical limb ischemia (CLI) with foot lesions (consistent with Rutherford classes 5 or 6). Exclusion criteria were acute limb-threatening ischemia, trauma, non-atherosclerotic disease (e.g., arteritis), embolic disease, known hypercoagulable state or any acute cardiac disease (e.g., acute coronary syndrome, heart failure, unstable arrhythmias) or acute non-cardiac disease (e.g., severe sepsis, acute kidney disease, pneumonia) that may cause an increase of cardiac troponin.

### Definitions

For the definition of diabetes and CLI, the current standards and guidelines were followed. In short, diabetes was diagnosed according to Standards of Medical Care in Diabetes criteria (12); CLI was defined as the presence of chronic ischemic rest pain and/or non-healing ulcers or gangrene attributable to proven arterial occlusive disease (13). Artery disease was assessed through transcutaneous oxygen tension (<30 mm Hg) and imaging (Doppler examination and/or angiography). History of coronary artery disease (CAD) was defined as any documentation at the time of admission of previous myocardial infarction, coronary revascularization (surgical or percutaneous), and/or any imaging test indicating CAD (scintigraphy, CT, or magnetic resonance). The cardiovascular medical history of each patient was reviewed by an independent team. A multidisciplinary “foot team” including a diabetologist, a foot surgeon, a cardiologist, an interventional cardiologist, and a vascular surgeon were responsible for the diagnosis and management of CLI.

### Laboratory Assessment

Blood samples of all patients were collected at admission. High-sensitivity troponin T (hs-TnT) was measured with Elecsys Troponin T hs assay using a Cobas e601 analyzer (Roche Diagnostics, Mannheim, Germany). Upper reference limit (URL, 99^th^ percentile), limit of quantification, and limit of detection of the assay were 14, 13, and 5 ng/L, respectively. Further hs-TnT samples were collected in case of admission values above URL to detect myocardial injury. Blood count, lipid profile, glycated hemoglobin, and serum creatinine were measured with standardized methods in all patients. The estimated glomerular filtration rate (eGFR) was calculated with the Cockcroft–Gault formula.

### SPINACH Study Score

Few prognostic scores are available in the setting of CLI. Recently, a predictive model for mortality risk in CLI patients has been derived from the SPINACH (surgical reconstruction vs. peripheral intervention in patients with critical limb ischemia) study (7). The model did not include any serum biomarker and had a good predictive power at 2 years. To test if hs-TnT had an incremental value on this model, we analyzed its performance in our dataset.

### Follow-Up

Patients returned for surgical visits every 15 days until clinical stabilization of surgical site. Afterwards, they were visited every 2 months. During the visits, physical examination was performed, and patients were assessed for adverse events and compliance with medical therapy. Further examinations and tests were prescribed at the physician's discretion. All patients were followed for at least 1 year. Median follow-up was 981 [557–1325] days, with 267 (43%) patients reaching the 2-year timepoint.

### Endpoints

The primary endpoint was the major cardiovascular events (MACEs, defined as composite endpoint of all-cause death, myocardial infarction, and stroke) (14). The secondary endpoint was all-cause mortality. Source documents of adverse events were collected and reviewed by independent blinded reviewers from another institute (University Hospital of Ferrara) for the final adjudication.

### Statistical Analysis

Continuous variables are presented as mean ± SD or median [interquartile range] and categorical variables as counts and proportions (%). For continuous variables, the differences were compared between groups (CAD status) using the one-way ANOVA and the Kruskal–Wallis test for parametric and non-parametric data, respectively. Survival was estimated in the two groups of CAD for each outcome of interest from the Kaplan–Meier curves that were compared using the log-rank test. Cox proportional-hazards regression modeling was used to analyze the effect of several variables on MACE and all-cause mortality. All baseline variables were tested in univariate model, and those found to be significative (*p* < 0.05) were included in adjusted multivariate Cox regression analysis. Results are reported as hazard ratios with associated 95% CIs. The multicollinearity was examined using the variance inflation factor (VIF) and variables with VIF >5 were excluded by the same multivariable model. Variable selection was performed by a backward stepwise algorithm based on Akaike's information criterion minimization. Cross-validation was performed to validate the models obtained. The *p*-value related to the likelihood ratio was calculated, along with Harrel's C-index. Hs-TnT cut-offs were determined for each outcome of interest in the two CAD groups applying the change-point method to the survival models. Multivariable Cox regressions were performed with discretized troponin variable to test its predictive values and results are reported as hazard ratio and 95% CI. Survival estimates were obtained from the Cox regression model toward outcomes and CAD status. Predictive model's capability was assessed by receiver operating characteristic (ROC) analysis and area under the curve (AUC), sensibility, and specificity estimation. To evaluate the performance of the SPINACH study model in our cohorts, model discrimination capability was assessed using time-dependent AUC (AUC-tdROC). Moreover, we added the troponin as predictor and assayed the model discrimination capability together with Bayesian information criterion (BIC) variation. The analysis was performed with R version 3.5.1 (R Foundation for Statistical Computing, Vienna, Austria).

## Results

### Patients' Characteristics

Between June 2015 and December 2017, 723 diabetic patients with critical limb ischemia and foot lesions were admitted to our Unit. One-hundred and five patients were not enrolled for the presence of exclusion criteria: 8 had non-atherosclerotic disease, 29 acute limb-threatening ischemia, 10 known hypercoagulable state, 24 concomitant acute cardiac conditions, 19 did not give informed consent, and 15 did not guarantee long-term follow-up. Thus, 618 patients represented the study population of the present analysis (Table 1). All patients underwent lower limb angiography and 597 (97%) performed percutaneous transluminal angioplasty (PTA). Five-hundred forty-nine (89%) patients received concomitant surgical treatment for diabetic foot lesions. Overall, 270 (43.7%) patients showed an established CAD diagnosis (50 previous MI, 217 previous PCI/CABG, 36 positive imaging tests). On the contrary, 348 (56.3%) patients did not have a CAD diagnosis. As expected, male sex, heart failure, atrial fibrillation, and chronic kidney disease were more frequent in the established CAD diagnosis group, while PAD status did not differ between groups.

**Table 1:** Characteristics of the study population.

	**Entire study population (*n* = 618)**	**Established CAD diagnosis (*n* = 270)**	**No CAD diagnosis (*n* = 348)**	***P***
Age, years	73 [65–80]	73 [65–80]	73 [65–80]	0.62
Male sex, no. (%)	445 (72.0)	223 (82.6)	222 (63.8)	<0.01
BMI, kg/m^2^	26.4 [23.9–30.3]	26.2 [23.9–29.7]	26.7 [24.2–30.4]	0.31
**Medical history, no. (%)**				
Hypertension	544 (88.0)	236 (87.4)	308 (88.5)	0.77
Dyslipidemia	473 (76.5)	219 (81.1)	254 (73.0)	0.02
Current smokers	57 (9.3)	23 (8.7)	34 (9.8)	0.73
Type 1 diabetes	23 (3.7)	8 (3.0)	15 (4.3)	0.51
Prior PCI/CABG	217 (35.6)	217 (82.8)	–	–
Chronic heart failure	78 (13.3)	50 (19.8)	28 (8.4)	<0.01
Atrial fibrillation	160 (26.1)	92 (34.6)	68 (19.5)	<0.01
Carotid artery disease	87 (14.7)	54 (21.3)	33 (9.7)	<0.01
Previous stroke	70 (11.4)	37 (13.9)	33 (9.5)	0.10
CKD	340 (55.0)	163 (60.4)	177 (50.9)	0.01
ESRD on dialysis	64 (10.4)	29 (10.8)	35 (10.1)	0.88
COPD	62 (10.1)	34 (12.6)	28 (8.1)	0.08
**Laboratory data**				
Hemoglobin, g/dl	11.9 ± 1.8	11.8 ± 1.7	11.9 ± 1.9	0.66
White blood cells, 10^3^/μl	8.9 [7.1–11.0]	8.9 [7.1–11.1]	8.9 [7.2–10.8]	0.81
Platelets, 10^3^/μl	274.2 ± 104.9	265.5 ± 100.5	280.8 ± 107.4	0.07
eGFR, ml/min	56.4 [37.6–79.7]	52.6 [36.6–74.7]	59.4 [39.8–84.0]	0.01
HDL, mg/dl	39.0 [31.0–49.0]	38.0 [31.0–45.0]	41.0 [31.0–52.0]	0.02
Triglycerides, mg/dl	135.7 ± 67.8	134.2 ± 70.1	137.0 ± 66.0	0.61
LDL, mg/dl	77.4 [56.1–102.2]	70.8 [50.6–96.2]	81.0 [62.4–106.3]	<0.01
Hemoglobin A_1c_, mmol/mol	56.0 [46.0–68.0]	56.0 [46.0–68.3]	56.0 [47.8–68.0]	0.96
CRP, mg/dl	1.10 [0.40–4.70]	1.20 [0.40–4.60]	1.10 [0.40–4.90]	0.95
Albumin, g/dl	3.58 [3.14–3.88]	3.59 [3.14–3.87]	3.58 [3.15–3.89]	0.82
**PAD status, no. (%)**				
Previous PTA	257 (41.9)	125 (46.6)	132 (38.2)	0.04
Previous amputation	211 (34.2)	98 (36.3)	113 (32.6)	0.38
ATK artery disease	362 (59.8)	164 (62.6)	198 (57.7)	0.26
BTK artery disease	572 (93.0)	249 (92.9)	323 (93.1)	1.00
Rutherford category				0.93
5	492 (79.6)	214 (79.3)	278 (79.9)	
6	126 (20.4)	56 (20.7)	70 (20.1)	
Transcutaneous oxygen tension, mmHg	15 [5–19]	12 [4–17]	17 [5–21]	0.17
Osteomyelitis	258 (44.8)	113 (46.3)	145 (43.7)	0.59
**Medical therapy, no. (%)**				
Oral anticoagulants	112 (18.2)	61 (22.7)	51 (14.7)	0.01
Aspirin	563 (91.2)	248 (92.2)	315 (90.5)	0.57
Clopidogrel	576 (93.4)	253 (94.1)	323 (92.8)	0.63
ACEi/ARBs	320 (51.9)	140 (52.0)	180 (51.7)	1.00
Beta-blockers	316 (51.2)	176 (65.4)	140 (40.2)	<0.01
Statins	488 (79.2)	220 (81.8)	268 (77.2)	0.19
Insulin therapy	467 (75.9)	214 (79.9)	253 (72.9)	0.06
Insulin units (IU/die)	32 [16–50]	32 [18–50]	32 [12–50]	0.60
Oral antidiabetic drugs	160 (26.0)	57 (21.2)	103 (29.7)	0.02

*ACEi, angiotensin-converting enzyme inhibitors; ARBs, angiotensin receptor blockers; ATK/BTK, above/below the knee; BMI, body mass index; CABG, coronary artery bypass graft; CAD coronary artery disease; CKD, chronic kidney disease; COPD, chronic obstructive pulmonary disease; CRP, C-reactive protein; eGFR, estimated glomerular filtration rate; ESRD, end-stage renal disease; HDL, high-density lipoprotein; LDL, low-density lipoprotein; PAD, peripheral artery disease; PCI, percutaneous coronary intervention; PTA, percutaneous transluminal angioplasty*.

### Troponin Values

Median value of hs-TnT in the overall population was 31 (20–59) ng/L. Overall, 525 (85%) patients had values above the URL. As expected, patients on hemodialysis had a significantly higher level of hs-TnT (87 vs. 30 ng/L, *p* < 0.01). The difference in troponin level between Rutherford categories were not significant (*p* = 0.24). Patients with established CAD diagnosis showed hs-TnT values significantly higher as compared with those without CAD diagnosis (39 vs. 29 ng/L, *p* < 0.01).

### Major Cardiovascular Events

**Figure 2:**
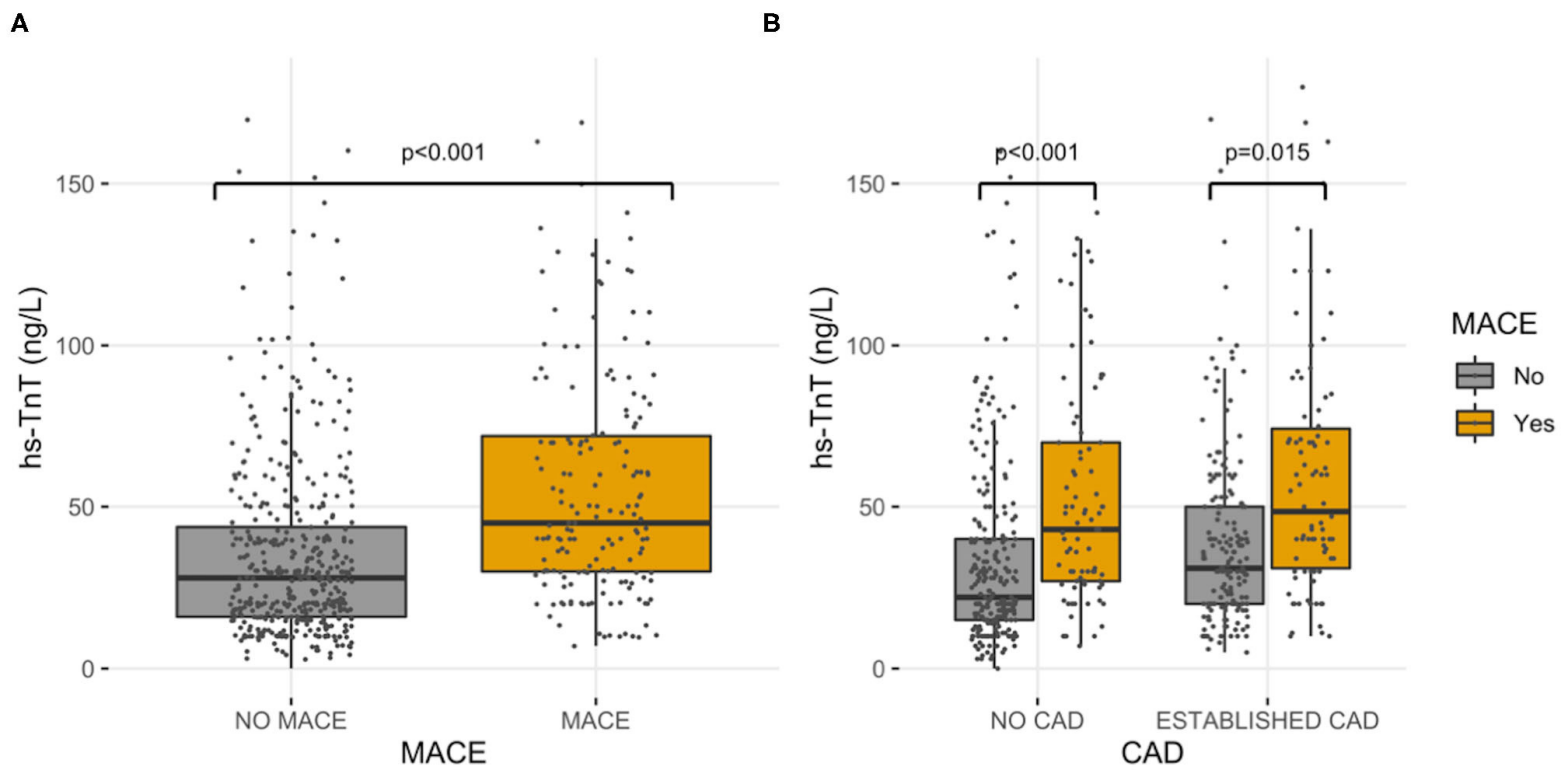
High-sensitivity cardiac troponin T values. Hs-TnT values, grouped by MACE, in the entire study population **(A)** and in established CAD and no CAD diagnosis sub-cohorts **(B)**.

During the follow-up, 179 (29%) patients experienced a major cardiovascular event (MACE). Overall, 155 (25%) patients died; 33 (5.3%) had a myocardial infarction, and 4 (0.6%) a cerebrovascular accident. The cause of death was cardiovascular in 48 patients (31% of cases). The values of hs-TnT at the admission of the index hospitalization were higher in those suffering MACE vs. those without MACE (75 vs. 40 ng/L, *p* < 0.001) (Figure 2A). Change point method analysis identified a hs-TnT cut-off of >40 ng/L for the prediction of MACE (AUC 0.712, 95% CI 0.657–0.756, sensitivity 93.7%, specificity 24.5%). After correction for potential confounding factors (Table 2), hs-TnT emerged as an independent predictor of MACE (HR 2.440, 95% CI 1.706–3.489, *p* < 0.001). As expected, the occurrence of MACE was higher in patients with established CAD (*n* = 90, 33%) as compared with those without CAD diagnosis (*n* = 89, 26%) (*p* = 0.04).

**Table 2:** Univariate and multivariable Cox regression for the prediction of MACE.

	**Univariate**		**Multivariable**	
	**HR (95% CI)**	***P***	**HR (95% CI)**	***P***
**Entire study population (*****n*** **=** **618)**				
BMI	0.745 (0.603–0.921)	0.007		
eGFR	0.475 (0.375–0.602)	<0.001	0.604 (0.470–0.778)	<0.001
Chronic heart failure	2.168 (1.487–3.162)	<0.001		
Hemoglobin	0.684 (0.541–0.863)	0.001		
Hemoglobin A1c	0.760 (0.615–0.939)	0.011		
CRP	1.145 (1.034–1.267)	0.009		
Albumin	0.770 (0.624–0.95)	0.015		
Insulin units	0.726 (0.581–0.907)	0.005		
Atrial fibrillation	1.996 (1.458–2.731)	<0.001	1.684 (1.207–2.351)	0.002
ESRD on dialysis	3.077 (2.128–4.450)	<0.001		
ACEi/ARBs	0.636 (0.470–0.861)	0.003		
Statins	0.678 (0.487–0.943)	0.021		
Osteomyelitis	0.702 (0.508–0.969)	0.032		
Stroke	2.074 (1.397–3.080)	<0.001		
WBC	1.162 (1.067–1.266)	<0.001		
COPD	2.058 (1.344–3.152)	<0.001		
**Established CAD (*****n*** **=** **270)**				
eGFR	0.580 (0.424–0.794)	<0.001		
Chronic heart failure	1.924 (1.171–3.163)	0.010		
Hemoglobin A1c	0.727 (0.542–0.976)	0.034		
Insulin units	0.672 (0.487–0.928)	0.016	0.672 (0.465–0.972)	0.035
Previous stroke	2.135 (1.277–3.571)	0.004	2.012 (1.127–3.592)	0.018
ESRD on dialysis	2.942 (1.723–5.022)	<0.001		
COPD	2.209 (1.261–3.873)	0.006	2.773 (1.465–5.249)	0.002
Statins	0.583 (0.361–0.943)	0.028		
**No CAD diagnosis (*****n*** **=** **348)**				
eGFR	0.417 (0.295–0.588)	<0.001	0.531 (0.362–0.781)	0.001
Chronic heart failure	2.337 (1.293–4.225)	0.005		
Hemoglobin	0.635 (0.462–0.873)	0.005		
WBC	1.169 (1.078–1.268)	<0.001		
HDL	0.707 (0.521–0.958)	0.025		
Dyslipidemia	1.636 (1.066–2.511)	0.024		
CRP	1.204 (1.052–1.378)	0.007		
Albumin	0.666 (0.503–0.883)	0.005		
Atrial fibrillation	2.798 (1.808–4.329)	<0.001	3.166 (1.704–5.882)	<0.001
ESRD on dialysis	3.170 (1.902–5.283)	<0.001		

*ACEi, angiotensin-converting enzyme inhibitors; ARBs, angiotensin receptor blockers; BMI, body mass index; CAD coronary artery disease; COPD, chronic obstructive pulmonary disease; CRP, C-reactive protein; eGFR, estimated glomerular filtration rate; ESRD, end-stage renal disease; HDL, high-density lipoprotein; WBC, white blood cells*.

### Patients With Established CAD Diagnosis

**Figure 3:**
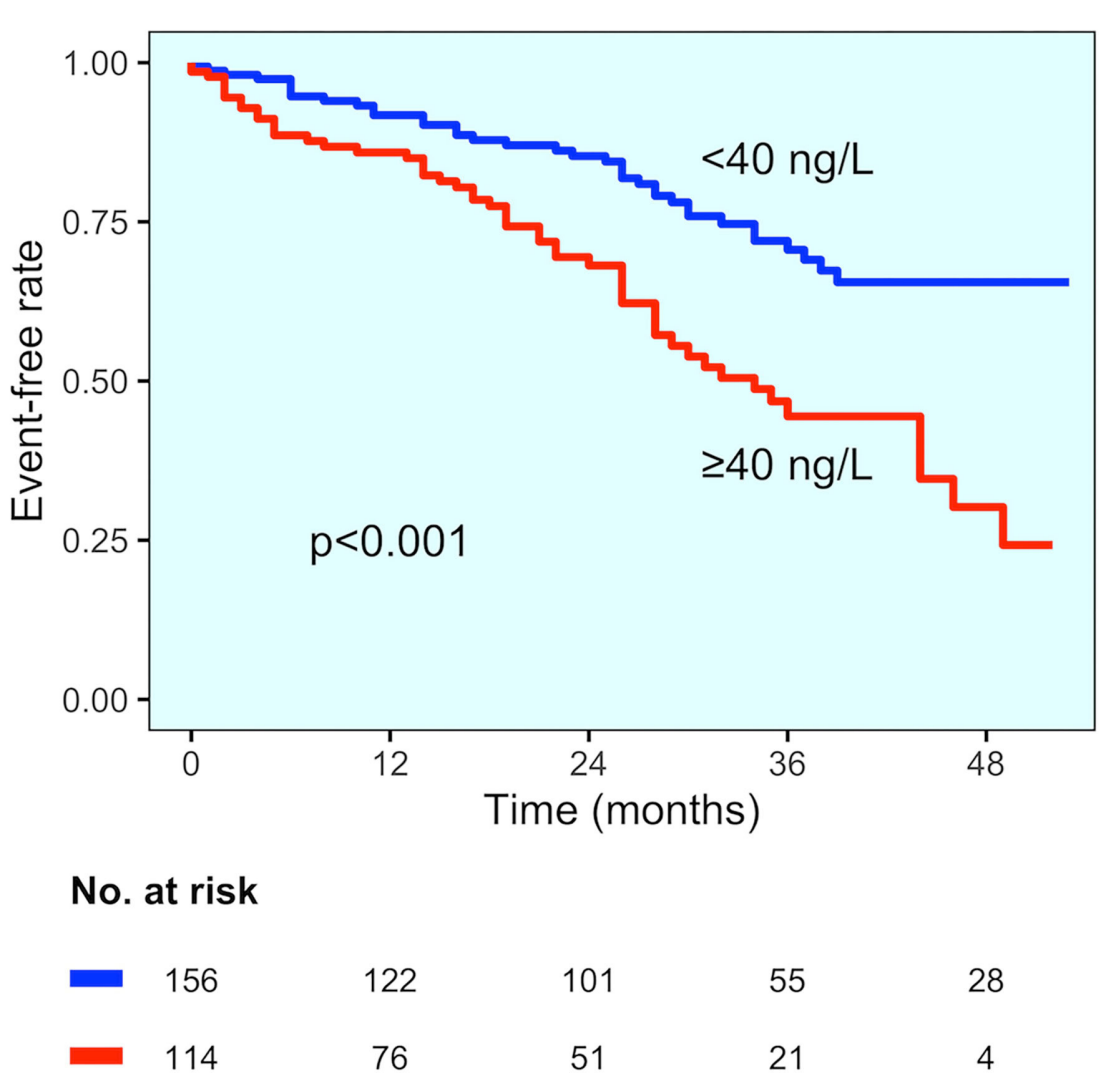
MACE incidence in the established CAD diagnosis group. Kaplan–Meier curves for MACE in patients with established CAD diagnosis, stratified by hs-TnT cut-off (40 ng/L).

In the group of patients with established CAD, those experiencing MACE had higher hs-TnT values compared to those without MACE (78 vs. 45 ng/L, *p* = 0.015) (Figure 2B). Even for this group, the change point method analysis identified a hs-TnT cut-off of >40 ng/L as that having the best predictive accuracy for MACE (AUC 0.711, 95%CI 0.694–0.824, sensitivity 91.3%, specificity 30.8%). Patients with established CAD and showing hs-TnT ≥40 ng/L (*n* = 114, 42%) had significantly higher MACE rate compared to those with a value <40 ng/L (46 vs. 24%, *p* < 0.001, respectively) (Figure 3). After correction for potential confounding factors (Table 2), hs-TnT ≥40 ng/L remained associated with a 2.9-time increase in the risk of MACE (HR 2.951, 95%CI 1.693–5.146, *p* < 0.001).

### Patients Without CAD Diagnosis

**Figure 4:**
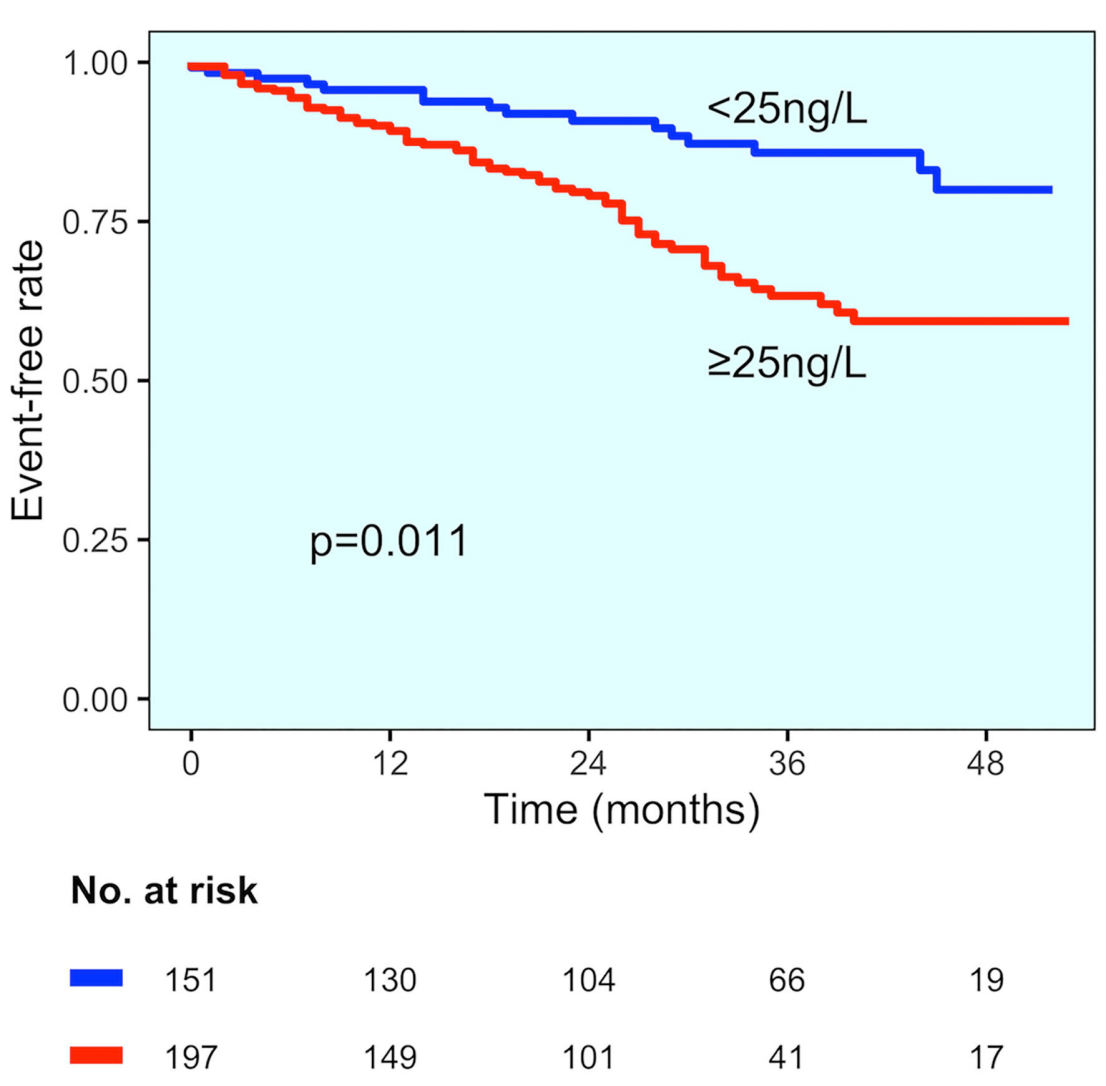
MACE incidence in the no CAD diagnosis group. Kaplan–Meier curves for MACE in patients with no CAD diagnosis, stratified by hs-TnT cut-off (25 ng/L).

In the group of patients without CAD, those experiencing MACE showed higher hs-TnT values than those without MACE (73 vs. 37 ng/L, *p* < 0.001) (Figure 2B). Change point method analysis identified the best cut-off at hs-TnT levels of ≥25 ng/L for prediction of MACE (AUC 0.725, 95% CI 0.698–0.827, sensitivity 95.4%, specificity 30.4%). The occurrence of MACE was higher in patients without CAD diagnosis and hs-TnT ≥25 ng/L compared with those with a value of <25 ng/L (37 vs. 25%, *p* < 0.001, respectively) (Figure 4). At multivariable analysis, after correction for confounding factors (Table 2), hs-TnT ≥ 25 ng/L remained an independent predictor of MACE (HR 2.291, 95% CI 1.21–4.337, *p* < 0.011).

### All-Cause Mortality

**Figure 5:**
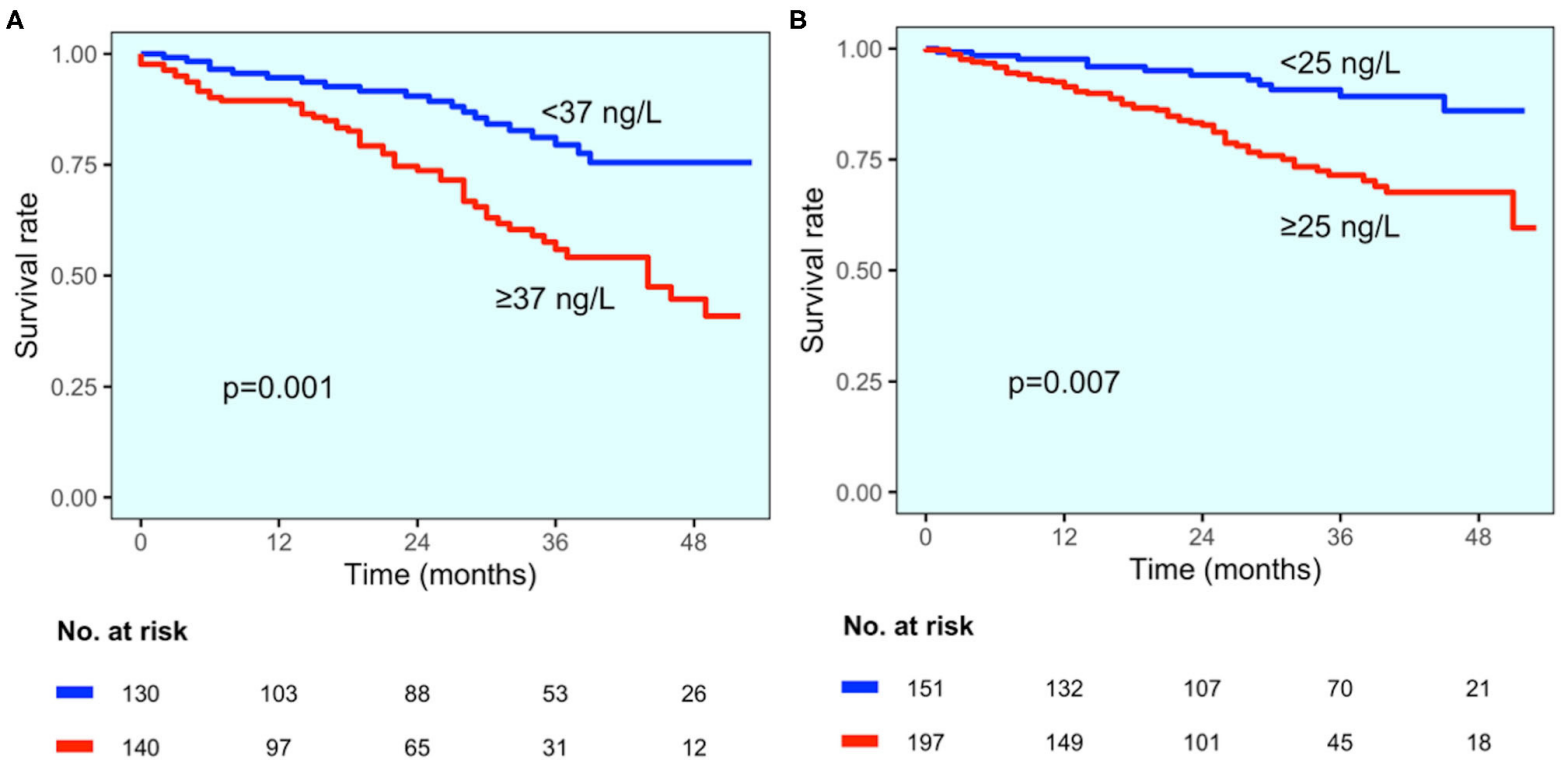
All-cause mortality according to CAD status. Kaplan–Meier curves for all-cause mortality for patients with established CAD diagnosis (**A**) and those without CAD diagnosis (**B**), stratified by hs-TnT cut-off values (37 and 25 ng/L, respectively).

The values of hs-TnT were significantly higher in patients who died (in the group with established CAD hs-TnT levels were 79 vs. 46 ng/L in alive patients, *p* = 0.033; in the group without CAD diagnosis 77 vs. 36 ng/L, *p* < 0.001, respectively). The best cut-offs identified by change point method analysis were 42 ng/L for the entire study population and 37 and 25 ng/L for patients with and without CAD diagnosis, respectively. Survival curves in study groups stratified according to best cut-offs are reported in Figure 5. At multivariable analysis, hs-TnT ≥37 and ≥25 ng/L were associated with a 2.9-fold and 2.8-fold increase in the risk of mortality (HR 2.899, 95% CI 1.548–5.431, *p* < 0.001 and HR 2.756, 95% CI 1.322–5.745, *p* = 0.007, respectively).

### Incremental Value of hs-TnT for the Prediction of All-Cause Mortality

The SPINACH study score confirmed its prognostic ability in the study population (AUC-tdROC 0.632). The addition of hs-TnT in the model (cut-off 42 ng/L) significantly improved the ability to predict all-cause mortality (Table 3). This finding is consistent only for the group without CAD diagnosis, while for patients with established CAD diagnosis there was only a trend for improvement (Table 3).

**Table 3:** Prediction improvement with the addition of hs-TnT to SPINACH study score.

	**AUC-tdROC**		***P***	**ΔBIC**
	**SPINACH**	**SPINACH + hs-TnT**		
Entire study population	0.632^*^	0.739^*^	<0.001	−34.576
Established CAD diagnosis	0.699^*^	0.763^*^	0.089	−16.558
No CAD diagnosis	0.613^*^	0.740^*^	<0.001	−27.141

*AUC-tdROC, area under the curve–time-dependent receiver operating characteristic; BIC, Bayesian information criterion; CAD, coronary artery disease*.

* *Wilcoxon test P-value <0.01*.

## Discussion

The main findings of our analysis are as follows:

1. Diabetic patients with CLI and foot lesions frequently have hs-TnT higher than URL at admission.
2. Those with established CAD diagnosis have higher hs-TnT than those without CAD.
3. Specific cut-offs, according to CAD status, predict MACE and mortality independently from other confounders.
4. hs-TnT improves the predictive power of the SPINACH study score.

The predicting role of hs-TnT in PAD patients has already been described, but only a few studies have focused on critical limb ischemia (CLI) (15, 16). Linnemann and colleagues analyzed non-high sensitivity troponin in a consecutive cohort of more than 1000 patients with PAD at different stages, from intermittent claudication (60%) to CLI (40%) (11). Only 290 (28%) had foot lesions. There was a wide different distribution of troponin through the stages: 60% of patients with foot lesions had detectable cTnT (greater than URL), vs. 15% or less in patients without foot lesions or intermittent claudication. The study showed a predictive role for detectable cTnT independently from PAD stage. Our study confirms and expands these findings.

First, we analyzed troponin in a sizeable homogenous cohort of patients with CLI and foot lesions, an under-examined sub-population at high risk of major cardiovascular events. Second, compared with past studies, we used a high-sensitivity assay. Patients with hs-TnT higher than URL were 85%, which means that using the URL as cut-off might be limiting. Indeed, we found that the cut-offs with the best predictive power for MACE and mortality were about three-fold higher than URL. Third, we performed the analysis dividing our population according to coronary artery disease (CAD) diagnosis, a condition known to influence troponin values. Hs-TnT was found to be an independent predictor of MACE and mortality in both groups, with higher cut-offs in patients with established CAD diagnosis. Fourth, hs-TnT showed additional predictive value on top of the SPINACH study score. The latter is a complex score for mortality prediction mixing several clinical variables (age, impaired mobility, BMI, renal failure, heart failure, and the WIfI Wound grade). It was derived in a population of 520 Japanese CLI patients but has never been validated externally. Its performance was discrete in our population (AUC 0.632), and we found additional benefit from the inclusion of hs-TnT (AUC 0.739), indirectly confirming its ability to stratify patients. Risk scores are useful in clinical practice, especially to help in the management of complex patients as those with CLI. Hs-TnT is a single variable with good ability in stratifying risk and should be taken into account for the development of new multivariable models.

Our findings are clinically relevant and can immediately be implemented in daily practice for stratification of CV risk in diabetic CLI patients with foot lesions. Troponin is a widespread biomarker, easy to assay, and may help to recognize diabetic CLI patients in which preventive strategies should be more aggressive. For patients with already established CAD group, several options for secondary prevention are available. A sub-analysis of the PEGASUS-TIMI 54 (Prevention of Cardiovascular Events in Patients with Prior Heart Attack Using Ticagrelor Compared to Placebo on a Background of Aspirin–Thrombolysis in Myocardial Infarction 54) trial show that the addition of ticagrelor to aspirin reduces recurrent ischemic events in diabetic patients with previous MI (17). The same was shown in the THEMIS-PCI (The Effect of Ticagrelor on Health Outcomes in DiabEtes Mellitus Patients Intervention Study—Percutaneous Coronary Intervention) trial for diabetic patients with stable CAD and previous PCI but without previous MI (18). Another sub-study of the PEGASUS-TIMI 54 trial showed that PAD patients had a greater benefit compared with non-PAD patients when ticagrelor was added to aspirin (19). A sub-study of the EUCLID (Examining Use of Ticagrelor in Peripheral Artery Disease) trial showed a reduction of MACE with ticagrelor vs. clopidogrel in PAD patients with previous coronary stenting (6). The COMPASS (Cardiovascular Outcomes for People Using Anticoagulation Strategies) trial showed the superiority of a strategy of aspirin plus low-dose rivaroxaban in patients with stable CAD and/or PAD to improve cardiovascular outcomes (20).

The recently studied sodium-glucose co-transporter-2 (SGLT2) inhibitors, empagliflozin and dapagliflozin in EMPA-REG OUTCOME (Empagliflozin Cardiovascular Outcome Event Trial in Type 2 Diabetes Mellitus Patients) and DECLARE–TIMI 58 (Dapagliflozin Effect on Cardiovascular Events–Thrombolysis in Myocardial Infarction 58) trials, respectively, reduced the rate of cardiovascular death of diabetic patients compared with placebo (21, 22). The same was true for canagliflozin in the CANVAS (Canagliflozin Cardiovascular Assessment Study) trial, but an increase of amputations was observed (23). Proprotein convertase subtilisin/kexin type 9 (PCSK9) inhibitors alirocumab and evolocumab in ODYSSEY OUTCOME (Evaluation of Cardiovascular Outcomes After an Acute Coronary Syndrome During Treatment With Alirocumab) and FOURIER (Further Cardiovascular Outcomes Research with PCSK9 Inhibition in Subjects with Elevated Risk) trials, respectively, reduced MACE when added to statin therapy in patients with cardiovascular disease (24, 25).

In patients without a diagnosis of CAD, the clinical question is if to perform or not an active search of CAD. A “presumption of guilt” is reasonable in these patients, considering the high probability of CAD-PAD co-existence. However, an indiscriminate screening for CAD could be associated with unappropriated interventions and its cost-effectiveness could be questionable. On the contrary, we may speculate that such screening could be helpful in a selected subgroup at higher risk (as identified by hs-TnT). Coronary CT could be a first-step non-invasive screening examination. Another more invasive option is to program a coronary angiography or, better, perform it during the same session of the angiography of the lower limb. In both cases, a functional evaluation should be mandatory before proceeding with revascularization. However, whether hs-TnT would be effective in such screening will need to be ascertained in further studies. Regarding medical therapy, patients with unknown CAD status may benefit from SGLT2 inhibitors and low-dose rivaroxaban as PAD was an inclusion criterion *per se* in the trials, regardless of CAD diagnosis. The same is true for evolocumab, as, in a sub-analysis of FOURIER trial, PAD patients treated with evolocumab had less absolute risk than patients with coronary or cerebrovascular disease (26). All the available information, taken together, suggest a role for hs-TnT to stratify the risk of CLI patients with foot lesions independently from the previous diagnosis of CAD as innovative effective strategies are available to reduce the high risk of events to which these patients are exposed.

### Study Limitations

The present study has limitations. First, hs-TnT cut-offs were not validated in external cohorts. Generalizability remains a possible issue and should be externally validated. To this purpose, a prospective validation study might be implemented to support cut-offs' general applicability. Second, even though in many cases multiple hs-TnT samples were collected, only the first one (i.e., the one collected at admission) was considered in the analysis, to have values at the same standardized time point for all patients. Importantly, patients with acute coronary syndrome at admission were excluded. Third, the study was focused on patients with CLI and foot lesions. Cut-offs may vary according to PAD severity. Finally, our study was single center.

## Conclusions

In diabetic patients with critical limb ischemia and foot lesions, it is common to find high-sensitivity troponin T higher than URL. Depending on the presence of coronary artery disease diagnosis, specific hs-TnT cut-offs predict major cardiovascular events and mortality and may help to improve the patient's risk stratification.

## Data Availability Statement

The raw data supporting the conclusions of this article will be made available by the authors, without undue reservation.

## Ethics Statement

Ethical review and approval was not required for the study on human participants in accordance with the local legislation and institutional requirements. The patients/participants provided their written informed consent to participate in this study.

## Author Contributions

PC, LD, ET, DB, RF, and GC have made substantial contributions to the conception and design of the work. AC, GS, SB, DB, GP, FV, and EG have made substantial contributions to the acquisition and interpretation of data. PC, LD, MM, RP, and GC have made substantial contributions to the analysis and interpretation of data. PC, LD, AC, GS, ET, RP, DB, RF, and GC have drafted the work or substantively revised it. All authors read and approved the final article. PC accepts full responsibility for the work and the conduct of the study, had access to the data, and controlled the decision to publish.

## Conflict of Interest

The authors declare that the research was conducted in the absence of any commercial or financial relationships that could be construed as a potential conflict of interest.
